# From supramolecular chemistry to the nucleosome: studies in biomolecular recognition

**DOI:** 10.3762/bjoc.12.175

**Published:** 2016-08-17

**Authors:** Marcey L Waters

**Affiliations:** 1 Department of Chemistry, CB 3290, University of North Carolina at Chapel Hill, Chapel Hill, NC 27599, USA

**Keywords:** α-helices, aromatic interactions, β-hairpin peptides, cation–π interactions, dynamic combinatorial chemistry, histone, molecular recognition in water, nucleosome, π–π-stacking, post-translational modification, supramolecular chemistry

## Abstract

This review highlights the author’s indirect path to research at the interface of supramolecular chemistry and chemical biology.

## Review

### Childhood influences

**Figure 1:**
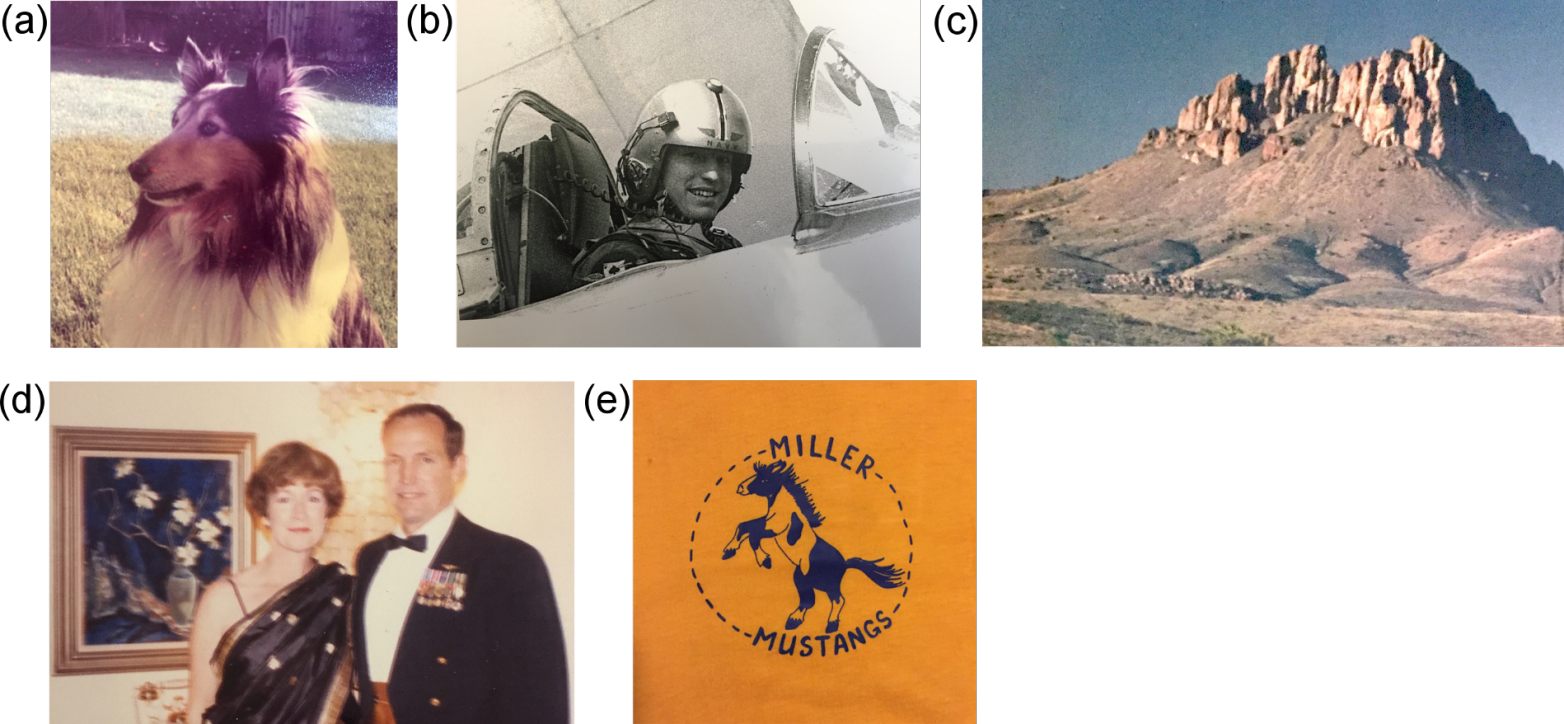
(a) Frodo the dog (copyright to MLW). (b) Ron Waters in an A6 in 1961 at the age of 26 (reproduced with permission from RLW). (c) A picture of Steeple Rock, near Duncan, AZ where we spent many mornings collecting geodes when visiting my grandparents (reproduced with permission from RLW). (d) Sue and Ron Waters in front of a painting by Sue Waters in 1983 (reproduced with permission from ASW and RLW). (e) My first successful artwork – the school logo I designed in elementary school (reproduced with permission from Miller Elementary School).

When thinking about how to start writing this review, it crossed my mind that the fact that I grew up with a dog named Frodo (Figure 1), named by my dad, says a lot about the environment in which I grew up (I read the Lord of the Rings at age 12 to learn why my dog was named that). Since I have already brought my dad into this, I will begin by saying a bit more about him and his influence on me. My dad is an intensely curious man who loves all things science. He started out as a geologist, but life took him in other directions, and he ended up as a Navy pilot and later an engineer. Nonetheless, my dad never lost interest in his first love, and so I learned a lot about rocks as a kid! I am the middle of three sisters, and we joke that my dad didn’t care if he had a son, as long as he had a scientist. My mom is an artist but followed a career path that was available to women at the time: she was a teacher before she had a family. Important for my story, I often overheard her commenting that women could do anything that men could do and that it was an outrage that women got paid less than men for the same work (and still is). Even at 5 or 6 years old, I remember getting angry about this myself and thinking, effectively, “I’ll show them”. So, between my own inherent interest in math and science (as is common among us, I asked for microscopes and chemistry sets for Christmas), strong encouragement from my dad, and a certain drive to prove something to the world (!) instilled by my mom, I set out on the trajectory that led me to where I am today (with a little help from some influential people along the way).

### The winding path to chemistry

Even so, it wasn’t a straight path to chemistry professor. In addition to my interest in science, I also loved to build and to draw and paint (my parents got me a real tool set when I was 6 or 7 and I promptly sawed into the picnic table in the backyard). In fact, my high school art teacher encouraged me to pursue art as a career, and for a time I considered architecture. And while early on chemistry was my favorite field of science, I was not inspired by it in High School or in general chemistry in college at UCSD (here’s how not to teach genchem: my textbook listed all compounds by their molecular formula, so, for example, acetic acid was C_2_H_4_O_2_. Thus, the fact that molecular structure has anything to do with reactivity was completely left out). I actually started out as a bioengineering major in college. However, after getting accepted to the impacted major (one in which only a subset of students is admitted through an application process), I realized I did not have a passion for it. I thought that genetics was interesting, so registered for both genetics and organic chemistry with the plan of being a biochemistry major. I had heard all of the dreadful stories about organic chemistry and actually went into the class with a bit of a sick curiosity (I had figured out in high school that I often like subjects that others dreaded, so I was not deterred by the dorm-room rumors of O-Chem). It turned out that, as is true with many organic chemists I know, I fell in love with the logic of organic chemistry (I cannot understate the influence of Professor Charles Perrin, who taught organic chemistry from a mechanistic perspective with beautiful clarity). I also suspect that the visual nature of the material appealed to me, as I inherited some degree of artistic aptitude from my mother and had always excelled at spatial relations like my dad. Thus, visualization of concepts like stereochemistry came easily to me, unlike many of my peers. I did pursue one semester of research in genetics, but at that point I found biology too vague for me; molecular level detail was what satisfied my curiosity. Indeed, it was only later once I felt I had a strong molecular understanding of molecular recognition principles that underpinned all of the cartoons of protein complexes that I turned back toward biology.

### On to graduate school in organometallic chemistry

Once I “found” organic chemistry, the path to graduate school was relatively direct. My TA, Rich Engler, encouraged me to pursue research, and I did so, joining the group of Professor Perrin, who had engaged me in organic chemistry in the first place. I had a penchant for physical organic chemistry (I wanted to know how things worked), so this was a good fit for me. I also participated in a summer NSF-REU program at Columbia University in Professor Ged Parkin’s group and got a taste of inorganic chemistry and all the fun of Schlenk line and glovebox techniques. I quickly decided that I wanted to attend graduate school and also very early on decided I wanted to be a professor. This was largely because I knew my research interests leaned toward the fundamental, but recognizing that there were few women faculty in the sciences in the late 1980’s to early 1990’s, there may have been a small part of me with something to prove, just like the 5-year old overhearing her mother’s conversations!

One interesting aside was the reaction of my parents (both of whom were the first in their families to go to college and whose childhoods bring up memories of rationing during World War II) when I told them I wanted to get a Ph.D. in chemistry. My mom said, “I don’t think we can afford that.” I explained that I could get paid to get a Ph.D. and she told me that I better check on that because that couldn’t be right. Every time I teach a big lecture class I make sure to tell my students about research opportunities, grad school, and getting paid to get a Ph.D., because I know there are still students out there just like me who didn’t come from a family of Ph.D.s and don’t know how the system works, and that you can still get paid to get an education!

**Figure 2:**
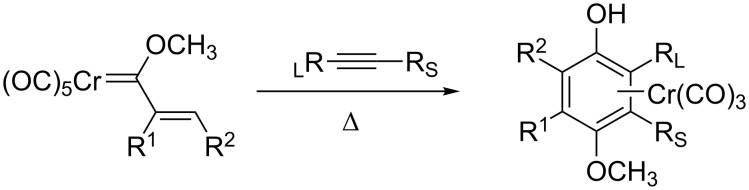
The Wulff–Dötz reaction.

With my research experiences in physical organic chemistry and inorganic chemistry, and with the boom in organometallic research at that time, I chose to pursue mechanistic organometallic chemistry for my Ph.D. at the University of Chicago in the group of Bill Wulff. Research in the group spanned organometallic methodology, asymmetric catalysis, total synthesis, and mechanistic studies. I opted for the latter and spent my Ph.D. studying the mechanism of the Wulff–Dötz reaction [1], while at the same time gaining a broad background in methodology and synthesis (Figure 2). I had a fantastic time in graduate school, with an advisor who loved to stand at the chalkboard and talk science for hours (one of my fondest memories). He was just the right mix of hands-on and hands-off for me, and knew how to motivate students through enthusiasm instead of pressure. As an example, we had group meetings on Friday mornings but no schedule. On Thursday afternoons, Bill would walk through the lab and talk to everyone about their latest results. Then on Friday morning, he would call on people to present their work. It didn’t take long to realize that he called on people with exciting new results, so everyone wanted to present at group meeting. Unlike many of my peers in graduate school, who left with a Ph.D. but no longer with a love of science, I made it through more enthusiastic than ever due to the positive mentorship I received. Reflecting on my own experience versus those of my peers in graduate school has had a significant impact on how I run my own group.

### A turn to bioorganic chemistry

**Figure 3:**
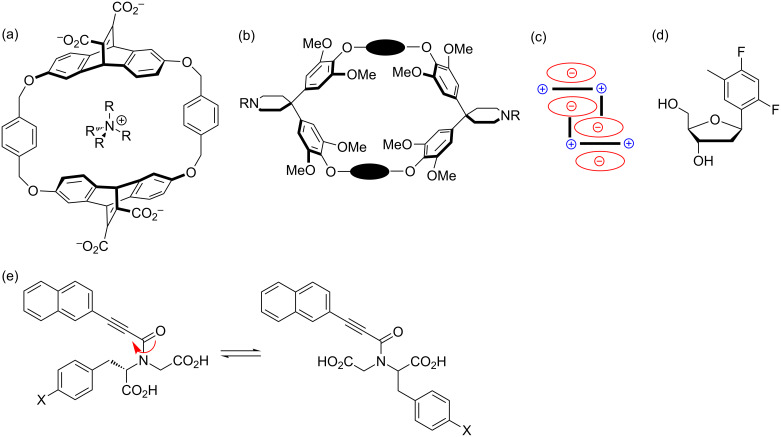
Work by others that inspired my interests. (a) Cyclophane receptors from Dennis Dougherty’s group in the late ‘80’s and early ‘90’s that demonstrated cation–π interactions [2]. (b) Cyclophane receptors from the Diederich group in the late ‘80’s and early ‘90’s that demonstrated the “nonclassical hydrophobic effect” [3]. (c) The Hunter-Sanders Model for π–π stacking from 1990 [4]. (d) Kool’s nonpolar isostere of thymidine from 1995 [5].(e) Gellman’s model for π–π stacking in aqueous solution [6].

An interesting thing happened while I was in graduate school. I found myself reading papers in a relatively young field: supramolecular chemistry. This interest did not simply spring forth on its own, however; the seeds were planted when I was an undergraduate. I took a graduate physical organic chemistry class from Jay Siegel, who was an assistant professor at the time. In his class, in addition to presenting the usual material, he covered recent published literature on molecular recognition that caught my attention, such as Dennis Dougherty’s work on cation–π interactions (Figure 3a) [2]. Thus, while at the time I was intent on studying organometallic chemistry, my interest in supramolecular chemistry increased the more I read through graduate school, and particularly molecular recognition in aqueous solution, which I viewed as the most challenging and most important medium for molecular recognition. This led to my decision to postdoc for Ron Breslow at Columbia University, who is known for biomimetic chemistry, but at the heart of his cyclodextrin-based enzyme mimics is molecular recognition in water. Breslow’s style was very different than Wulff’s, but he was also a very supportive, positive advisor. While I never had a female mentor, I never felt the need for one in these research groups (both departments had one woman on their faculty during my time in those departments).

### Starting out on my independent career – combining peptide chemistry and supramolecular chemistry

I learned a great deal of things during my postdoc and it was a great experience for me. However, one thing I learned was that I did not want to start out my independent career trying to design and synthesize a functional molecule (receptor, enzyme mimic, etc), only to find out after several months of synthesis that it did not function as planned! I wanted to utilize versatile chemistry that allowed me to synthesize and evaluate the compound of interest quickly and modify it rapidly for further mechanistic studies. This led me to become a peptide chemist! This was a risky move as an assistant professor to venture into a new field in which I had no established record. But I have always been one to follow my interests, and it worked out for me in the end.

I continued to be interested in aromatic interactions and their potential role in biology as a postdoc. Seminal work probing the electrostatic component of π–π stacking and edge-face aromatic interactions as well as cation–π interactions was being published at the time, as well as tantalizing suggestions about their relevance in biological structure and function. In particular, I was inspired by work of Dennis Dougherty [2], Francois Diederich [3], Sam Gellman [6], Eric Kool [5] and Jeremy Sanders [4] to name a few working in the area at the time (Figure 3). I was particularly interested in addressing whether aromatic interactions provided a degree of selectivity that is not possible with classic aliphatic, hydrophobic interactions, based on the electrostatic component of aromatic interactions.

In the late 1980’s and early 1990’s, much work was also done defining the factors that stabilize monomeric α-helices, including the role of noncovalent interactions such as salt bridges, as exemplified by the pioneering work by Baldwin [7–8] and Kallenbach [9]. Thus, when I started at UNC in 1999, I decided to investigate the use of α-helical scaffolds to investigate aromatic interactions, including π–π and cation–π interactions in aqueous solution. The goal was to develop biologically relevant model systems to study these interactions in aqueous solution and to gain insight into the nature of these interactions, their biological relevance, and also see if we could use them to influence structure and function. Peptides were very appealing because of the ease of synthesis and the ease of systematic variation and we published several papers using α-helical scaffolds [10–11]. However, one limitation of α-helices is that their folding is not two state, thus requiring indirect methods to measure the influence of a noncovalent interaction on folding.

**Figure 4:**
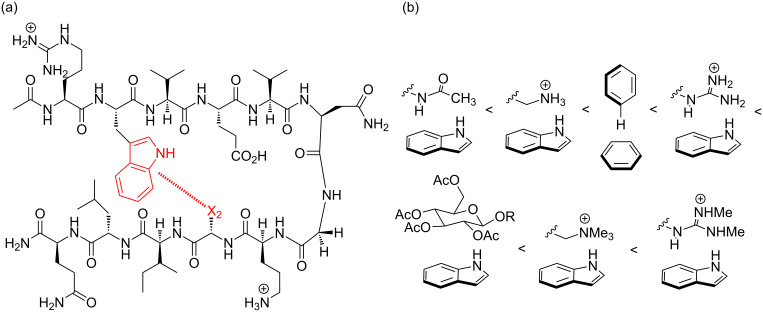
(a) Model β-hairpin for investigation of aromatic interactions. (b) Examples of noncovalent interactions studied, from weakest to strongest [16–32].

**Figure 5:**
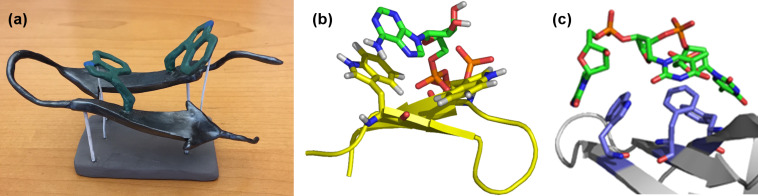
(a) A clay model of our WKWK peptide (aka “Saratide”) made by Jes Park, a former graduate student in the group (reproduced with permission from Jessica Park). (b) Computational model of Saratide bound to ATP. (c) Inspiration from Nature: an OB fold bound to ssDNA.

About that time, several papers had been published reporting the first monomeric, modestly folded, non-aggregating β-hairpins in aqueous solution [12–15]. My first student, Chad Tatko, read a paper by Gellman [15] on one of these early β-hairpins and suggested that we use it as a scaffold for exploring aromatic interactions. This was an attractive scaffold because a two-state approximation for folding was reasonable in most cases and the β-hairpin is far more amenable to NMR analysis than α-helices, which were usually characterized by Circular Dichroism (CD). Additionally, because the sidechains in β-hairpins interdigitate, they provide relatively isolated positions for evaluating noncovalent interactions, making them a superb model system. Chad and I set out on this course, which led to the publication of more than a dozen papers on a wide range of aromatic interactions in aqueous solution (Figure 4) [16–32]. At the same time, our model systems provided significant insight into the features that contribute to folding of β-hairpin peptides and β-sheets, an area that lagged decades behind the general understanding of α-helices. Beyond using β-hairpins as scaffolds for physical organic chemistry, we also developed some of the first functional β-hairpins that bound nucleotides and ssDNA, mimicking a class of β-sheet proteins, thus expanding on the sequence-structure-function paradigm with minimalist structures (Figure 5) [33–36]. More recently this work has been extended into catalytic β-hairpins that serendipitously utilize aromatic interactions to maximize catalysis [37–38]. Along the way, we had some fun naming the hairpins that had the most interesting properties, including Chadtide – our first model system (after Chad Tatko) [16], Saratide – which binds ATP (after Sara Butterfield) [33], Sarah–Zachtide – which investigates a carbohydrate–π interaction (after Sarah Kiehna and Zachary Laughrey) [27–28], Bobtide – which contains a cation–π interaction with KMe3 (trimethyllysine) and was the most stable β-hairpin reported at the time (after Robert Hughes) [22–23], and Alextide – for which folding can be turned on or off with post-translational modifications (after Alex Riemen) [32].

### Biological significance and a shift in focus

**Figure 6:**
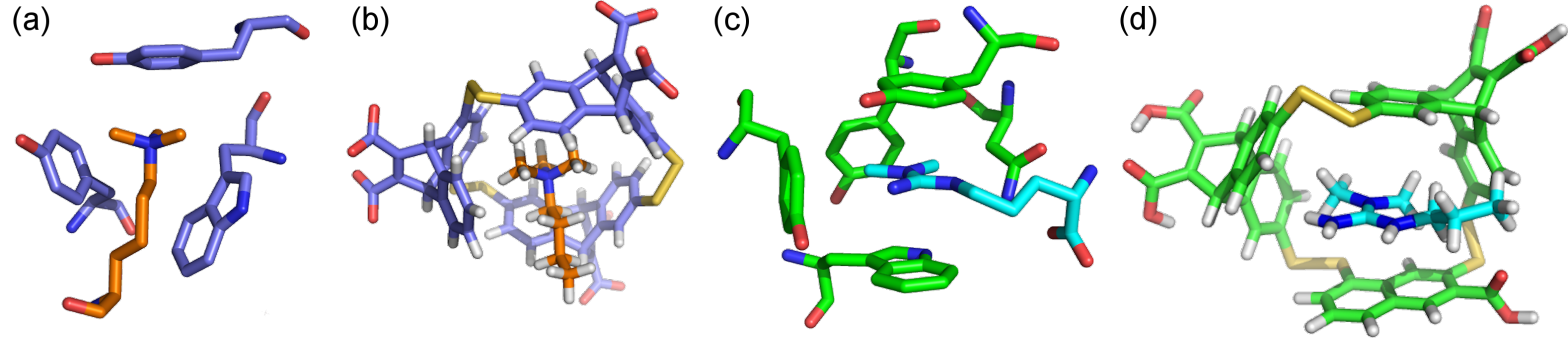
(a) Binding pocket of the *Drosophila* HP1 chromodomain (blue) bound to trimethyllysine (orange), PDB code: 1KNE [39]. (b) Computational model of a synthetic receptor, **A_2_N** (blue), bound to KMe3 (orange) [40] (c) Binding pocket of the SMN tudor domain (green) bound to asymmetric dimethylarginine (aDMA, cyan). (d) Computational model of a synthetic receptor, **A_2_D** (green), bound to aDMA (cyan) [41].

While studying aromatic interactions in β-hairpins in the early 2000’s, an important biological discovery was made: a crystal structure of a protein that binds to trimethyllysine (KMe3), an important post-translational modification involved in controlling gene expression, shows that it recognizes the trimethylammonium group via an aromatic cage (Figure 6a) [39]. This suggests that the binding is driven by cation–π interactions. We thus studied the influence of lysine methylation and the significance of the positive charge in our β-hairpin model systems [23–24], and then moved into studying the actual protein–peptide interaction as well, providing the first definitive evidence that cation–π interactions provided the dominant component to binding in this important class of interactions [26].

**Figure 7:**
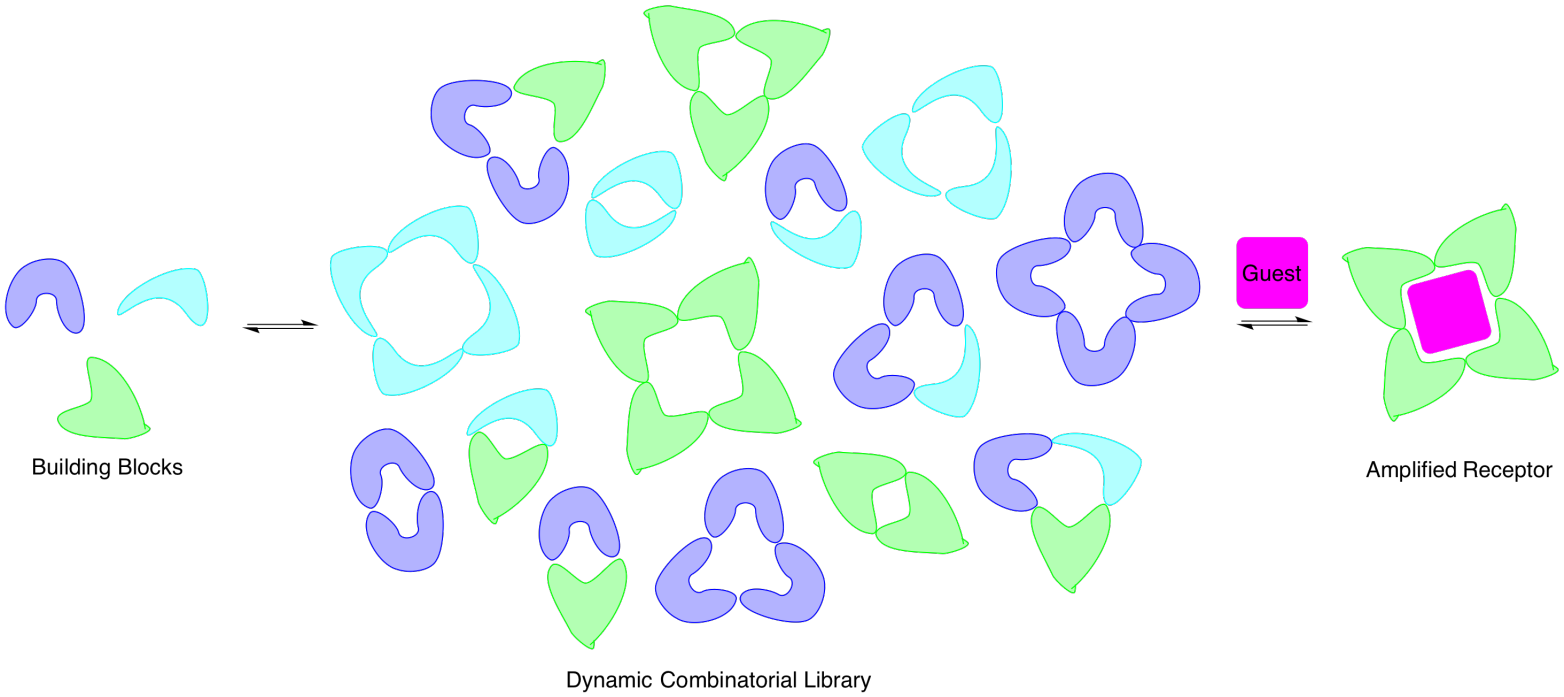
Dynamic combinatorial chemistry [41–42].

This work led to several important formal and informal collaborations with others doing research in the area of chromatin remodeling, and thus paved the way for a new direction in our research. I had been fascinated by the groundbreaking work of Jeremy Sanders and co-workers on dynamic combinatorial chemistry (DCC) while being a graduate student and postdoc (Figure 7) [42–43]. Like folded peptides that self-assemble into their functional state, DCC allows molecules to self-assemble in the presence of a template. Moreover, DCC is highly amenable to structure–function studies, since only a new monomer must be synthesized, rather than an entirely new receptor. With my interest in trimethyllysine provided a significant problem in which DCC seemed to be a promising solution. It turns out that the main tool for sensing protein post-translational modifications such as trimethyllysine are antibodies, but antibodies have significant limitations in this context, as they are too sequence specific. Thus, we aimed to develop synthetic receptors that would mimic the binding pockets of proteins to recognize trimethyllysine, but not the surrounding sequence. This turned out to be an ideal problem to address using DCC, and we have now developed a number of synthetic receptors for methylated lysine and arginine that have applications as sensors for these modifications (Figure 6).

### Lessons learned

As a child, my parents said that I “marched to my own drummer”. In my career I have continued to follow my interests wherever they have led me, which at points has meant effectively changing fields. This means that I always have new things to learn, which always keeps me interested. I look forward to seeing what is around the corner in the years to come.
